# Histone Tail Cleavage as a Mechanism for Epigenetic Regulation

**DOI:** 10.3390/ijms251910789

**Published:** 2024-10-08

**Authors:** Yonghwan Shin

**Affiliations:** Department of Biochemistry and Molecular Medicine, Norris Comprehensive Cancer Center, University of Southern California, Los Angeles, CA 90033, USA; shinyh81@gmail.com

**Keywords:** histone, histone modification, chromatin, cleavage

## Abstract

Histones are essential for DNA packaging and undergo post-translational modifications that significantly influence gene regulation. Among these modifications, histone tail cleavage has recently garnered attention despite being less explored. Cleavage by various proteases impacts processes such as stem cell differentiation, aging, infection, and inflammation, though the mechanisms remain unclear. This review delves into recent insights on histone proteolytic cleavage and its epigenetic significance, highlighting how chromatin, which serves as a dynamic scaffold, responds to signals through histone modification, replacement, and ATP-dependent remodeling. Specifically, histone tail cleavage is linked to critical cellular processes such as granulocyte differentiation, viral infection, aging, yeast sporulation, and cancer development. Although the exact mechanisms connecting histone cleavage to gene expression are still emerging, it is clear that this process represents a novel epigenetic transcriptional mechanism intertwined with chromatin dynamics. This review explores known histone tail cleavage events, the proteolytic enzymes involved, their impact on gene expression, and future research directions in this evolving field.

## 1. Introduction

Histones, being among the remarkably conserved proteins in eukaryotic organisms, orchestrate the structural organization of DNA into nucleosomes within the confines of the cellular nucleus [1,2,3,4,5]. These nucleosomes, constituted by approximately 146 nucleotide pairs enfolding around a core histone octamer comprised of H2A, H2B, H3, and H4, while tethered by H1 as a linker histone, represent the fundamental units in chromatin architecture [1,2,3,4,5]. Initially regarded as mere architectural scaffolds for DNA, recent investigations have illuminated the multifaceted roles of histones across various scientific domains [6].

**Figure 1:**
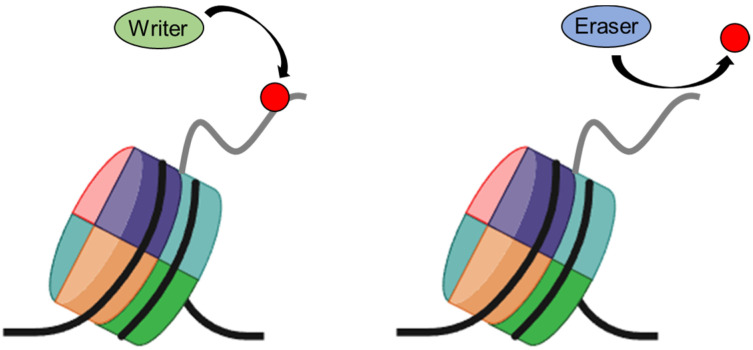
**Histone modifying enzymes involved in chromatin regulation.** The writer and eraser are groups of enzymes that act on histones; the writer adds small covalent modifications such as methyl and acetyl groups, while the eraser removes these modifications.

Recent research has unveiled the intricate involvement of histones in a spectrum of pathophysiological conditions, encompassing neurodegenerative ailments, oncogenesis, and inflammatory disorders such as sepsis [6]. Despite these explorations, the precise molecular underpinnings that connect histones to disease pathogenesis remain elusive. Post-translational modifications (PTMs), notably on histone tails, are pivotal in governing gene expression [7]. These diverse modifications, including acetylation, methylation, phosphorylation, and citrullination predominantly occurring on the N-terminal tails of histones, exert substantial influence on critical genomic processes such as transcription, DNA replication, and repair [8,9,10,11,12,13,14,15,16]. For example, the acetylation of lysine residues neutralizes their positive charge, weakening histone-DNA interactions and thereby facilitating access for transcription factors. [9,17]. The collective repertoire of these PTMs, often encapsulated within the framework of the “histone code”, intricately governs the accessibility of packaged DNA, consequently regulating gene expression [14,18,19]. Histone modifications within chromatin operate in a coordinated and precise manner to regulate gene transcription. The dynamic alteration of chromatin histone modifications is controlled through both chemical and physical mechanisms involving core histones [20,21,22]. A typical chemical mechanism is the reversible enzymatic addition and removal of specific histone marks by histone-modifying enzymes, referred to as writers, and erasers (Figure 1). In contrast, a more drastic physical mechanism involves ATP-dependent chromatin remodelers, which facilitate the exchange of canonical histones with histone variants, although histone chaperones and other mechanisms can conserve histone marks after replacement, particularly during processes such as replication.

Although the roles of many PTMs are well-established, the significance of histone tail proteolysis remains enigmatic. Histone hydrolysis, an irreversible PTM facilitated by an abundance of lysine and arginine residues within core histones, involves proteolytic cleavage catalyzed by proteases [14,18,19]. In addition to the impact of histone cleavage on individual histones within the octamer, it is crucial to understand its effects on the structural stability of H2A-H2B dimers and H3-H4 tetramers, which are significant in chromatin dynamics during chromatin remodeling [23,24]. H2A-H2B dimers are known to be less stable, and their dissociation from the nucleosome, facilitated by histone cleavage, enhances DNA accessibility for transcription, replication, and repair. On the other hand, the highly stable H3-H4 tetramers maintain nucleosome integrity, and cleavage within these histones can disrupt the tetramer structure, leading to alterations in nucleosome stability and positioning. This disruption influences higher-order chromatin structure and dynamics, impacting chromatin accessibility and compaction, which are critical for gene expression regulation and DNA repair. Therefore, understanding the specific effects of histone cleavage on these subunits provides a comprehensive view of chromatin dynamics and emphasizes the importance of histone modifications in chromatin structure and function regulation.

The area of histone proteolytic cleavage represents a dynamically evolving domain within epigenetics. Despite its emerging significance, the precise biological role and implications of this process in modulating gene expression remain subjects of active inquiry. This review aims to comprehensively examine current insights into the mechanisms governing histone cleavage, exploring its biological significance, and delineating the spectrum of enzymes implicated in this process. Furthermore, it endeavors to propose potential mechanisms through which histone cleavage may intricately regulate gene expression, thereby shedding light on its broader regulatory implications.

## 2. Cleavage of Histones H2A

An identified protease, isolated from purified calf thymus chromatin, demonstrated specific targeting of histone H2A, was confirmed to be specific to H2A (Table 1), and was identified as an aspartic acid protease with a cleavage site at Asn90-Asp91 towards the C-terminus [25]. Subsequent investigations formally classified this enzyme as the H2A specific protease (H2Asp) [26,27]. This truncation pattern was evident not only in myeloid and lymphatic leukemia cells [28,29,30], but also during the induced differentiation of THP-1 promonocytes into macrophages via retinoic acid [31,32]. Speculation emerged regarding a potential association between H2Asp and neutrophil elastase [33], a crucial protease linked to neutrophil extracellular trap (NET) formation. It was later suggested that H2Asp might be a substrate of neutrophil elastase [33]. Reports indicated that under conditions of reactive oxygen species production, neutrophil elastase translocates to the nucleus, selectively degrading specific histones, and possibly inducing chromatin decondensation [33,34]. However, the precise biological implications of C-terminally truncated H2A in NET formation remain ambiguous.

Subsequent investigations underscored the significance of C-terminally truncated H2A (1-114aa) in cellular stress susceptibility, emphasizing its pivotal role in maintaining cellular homeostasis [35]. Furthermore, these studies delineated the importance of the H2A C-terminal tail in both in vivo and in vitro nucleosome stability and mobility, and its regulatory role in chromatin remodeling processes mediated by Imitation Switch (ISWI)-type remodelers. Notably, this tail was identified to act as a recognition module for histone H1, thereby significantly impacting chromatin dynamics. This emphasized the critical involvement of the H2A C-terminal tail (115-129aa) in stabilizing the nucleosomal core particle and orchestrating protein interactions that regulate chromatin dynamics [35].

A distinct protease, exhibiting specificity towards histone H2A and resembling an aspartic acid-like protease, has been identified in liver nuclear extracts of both young and adult chickens. Additionally, H2Asp activity was not restricted to chickens, but was also observed in liver nuclear extracts from fish, frogs, and mice, suggesting a conserved function among vertebrates [36]. This protease specifically generates a single truncated H2A product (H2AGlu91) through in vitro cleavage assays. Notably, both its expression and activity were exclusively observed in liver nuclear extract, suggesting a tissue-specific occurrence. However, the functional implications of truncated H2A and the precise characterization of this protease remain to be fully elucidated. A recent study also reported that Cathepsin L cleaves histone H2A during embryonic stem cell differentiation and the role of this cleavage in altering H2A modifications and nucleosome stability during cell fate commitment [37].

**Table 1:** Characterization of histone proteases.

Histone	Protease	Cleavage Site(s)	Biological Significance of Activity	Model	Reference
**H2A**	H2A-specific protease	Val114-Leu115	Unknown	Calf thymus	[25]
	Neutrophil elastase	Val114-Leu115	Neutrophil extracellular trap (NET) formation	Neutrophil	[31]
	Histone H2A specific protease (H2Asp)	Asn90-Asp91	Unknown	Chicken liver extract	[33]
	Cathepsin L	Leu23-Gln24	Embryonic stem cells (ESCs) differentiation	Mouse embryonic stem cells (mESCs)	[38]
**H2B**	Tryptase	Unknown	Mast cell differentiation	Mouse mast cells	[35,36]
**H3**	Tryptase	Unknown	Mast cell differentiation	Mouse mast cells	[35,36]
	Cathepsin L	Ala21-Thr22, Arg26-Lys27, Ala31-Thr32	Embryonic stem cells (ESCs) differentiation	Human embryonic stem cells (hESCs)	[37]
		Ala21-Thr22, Thr22-Lys23, Lys23-Ala24,	Embryonic stem cells (ESCs) differentiation	Mouse embryonic stem cells (mESCs)	[38]
		Ala24-Ala25, Arg26-Lys27, Lys27-Ser28			
	Yeast endopeptidase	Ala21-Thr22	Induced under nutrient deprivation and sporulation	Saccharomyces cerevisiae	[38]
	JMJD5	Lys9-Ser10	Induced under DNA damage	Human lung cancer cells	[39]
	Glutamate dehydrogenase	Lys23-Ala24, Lys27-Ser28	Unknown	Chicken liver extracts	[40]
	Unknown	Unknown	Unknown	Tetrahymena micronuclei	[41]
	FMDV 3C protease	Leu20-Ala21	Host cell transcription shutoff	Hamster kidney fibroblast cells	[42]
	MMP-9	Lys18-Gln19	Osteclastogenesis	Bone marrow macrophages	[43]
			Melanomagenesis	Human melanoma	[44]
			Colonic carcinogenesis	Human colon cancer cells	[45]
	Cathepsin D	Lys23-Ala24	Involution mammary gland	Mouse mammary gland	[46]
	Vacuolor protease B (PrB)	Lys23-Ala24	Unknown	Saccharomyces cerevisiae	[47]
	Granzyme A	Unknown	Staurosporine-induced cell death	Human B lymphoblastoid cell	[48]
**H4**	Granzyme A	Unknown	Staurosporine-induced cell death	Human B lymphoblastoid cell	[49]
	Trypsin and Chymotrypsin	Arg17-Arg19	Intestinal cell differentiation	Human colon cancer cells	[50]

## 3. Cleavage of Histones H2B

An enzyme responsible for the cleavage of H2B tails has been identified (Table 1), revealing the involvement of tryptase in removing the N-terminal tails of histone H2B [38,51]. Tryptase, predominantly localized in the cytoplasmic secretory granules of mast cells and known for its pro-inflammatory functions, surprisingly exhibited nuclear translocation during cell death, initiating the cleavage of core histones. Strikingly, tryptase-mediated truncation of histones H2B and H3 was evident during mast cell differentiation. Furthermore, the localization of tryptase to heterochromatin and the increased chromatin resistance to micrococcal nuclease in tryptase-deficient cells implied its role in modulating chromatin structure, favoring euchromatin formation over heterochromatin.

Subsequent studies further elucidated tryptase’s role, suggesting that its absence leads to the age-dependent accumulation of H2BK5ac, which is associated with the upregulation of markers indicative of non-mast cell lineages [51]. These observations propose a dual role for tryptase in modulating gene expression, contingent upon the chromatin state of mast cells.

## 4. Cleavage of Histones H3

The proteolytic cleavage of histone H3 has been a subject of intense investigation compared to other histones. Histone H3 contains multiple susceptible sites targeted by various proteases [6]. N-terminal cleavage of H3 has been observed across diverse cellular processes, including mouse embryonic stem cell (ESC) differentiation, viral infections, aging, yeast sporulation, senescence, DNA damage responses, osteoclastogenesis, and cancer development [39,40,43,52,53,54,55] (Table 1). H3 protease activity has been identified in Tetrahymena micronuclei, avian liver tissues, human ESCs, and mouse mast cells [38,41,42,54,56]. Two electrophoretically distinct forms of histone H3, H3F (fast migrating) and H3S (slow migrating), were reported in Tetrahymena micronuclei. H3F is derived from H3S through a regulated proteolytic event during cell growth and division, suggesting a regular, generation-specific H3 proteolytic cleavage during cell cycle progression [24].

Evidence from studies highlights the impact of viral infections on histone integrity. For instance, foot-and-mouth disease virus (FMDV) infection in BHK cells leads to notable histone H3 depletion and the emergence of a novel chromatin-associated protein (Pi) observed migrating amidst histones H2A and H4 on SDS-polyacrylamide gels [39]. Pi was identified as a truncated H3 variant lacking the 20 N-terminal residues, attributed to the activity of FMDV 3C protease, which plays a role in viral polyprotein maturation [57]. Reports suggest that the FMDV 3C protease hampers gene transcription through the process of H3 truncation [58]. Cathepsin L, a lysosomal protease with implications in extracellular matrix degradation, cancer, bone remodeling, cardiovascular disease, and immune modulation, has nuclear localization and reported nuclear functions [46,47,59,60,61,62]. Cathepsin L has been demonstrated to play a proteolytic role in histone H3 during mouse ESC differentiation, highlighting its involvement in epigenetic changes during differentiation. Cathepsin L was identified as the H3 N-terminal tail clipping protease, potentially modulated by covalent modifications such as H3K18ac or H3K27me2. These modifications may affect downstream effects by hindering CBX27-H3K27 methylation binding [52].

In oncogene-induced and replicative senescence, H3.3 is preferentially cleaved over H3.1, leading to transcriptional downregulation of cell cycle genes by removing H3K4me3, implicating H3.3 tail cleavage in silencing cell cycle-promoting genes [43]. In a yeast model, increased endopeptidase activity toward histone H3 was observed in cells transitioning to a stationary phase or sporulation, revealing histone H3 cleavage after alanine 21 by a serine protease. The absence of H3 tail cleavage impaired gene expression activation during the stationary phase and sporulation [40]. Vacuolar proteinase B (Prb1) demonstrated cleavage activity toward the histone H3 N-terminus in yeast, specifically cleaving between Lys23 and Ala24 [48]. Glutamate dehydrogenase in quail liver showed H3 tail cleavage during aging [48,60]. In the involuting mammary gland, Cathepsin D caused preferential cleavage between lysine 23 and alanine 24 in histone H3 upon nuclear translocation mediated by tyrosine nitration [63]. JMJD5, a Jumonji C (JmjC) domain-containing protein, mediated H3 N-tail cleavage at H3K9me1 under stress conditions such as DNA damage responses, potentially regulating gene transcription [47]. In mast cells, tryptase functions as a ‘clippase’ for histones H3 and H2B. Tryptase deficiency resulted in altered H2BK5ac levels without affecting H3 post-translational modifications, indicating the unresolved biological significance of histone H3 clipping in mast cells. In staurosporine (STS)-induced Raji cells, the histone H3 N-terminal tail was cleaved by granzyme A (GzmA) [44].

The previous findings implicated matrix metalloproteinase 9 (MMP-9) as the primary protease for histone H3 N-terminal tail cleavage (H3NT) during osteoclast differentiation, melanomagenesis, and colon cancer development [45,49,53]. Despite MMP-9’s known role as a secretory protein, biochemical studies revealed its nuclear accumulation during osteoclastogenesis. MMP-9 specifically cleaved H3K18-Q19 in vitro and in vivo, facilitated by H3K18 acetylation, orchestrated by p300/CBP, and necessary for gene activation during osteoclast differentiation. The role of MMP-9 in promoting melanoma development was demonstrated by facilitating H3 N-terminal tail cleavage (H3NT) proteolysis within the promoter and coding regions of pro-melanomagenic genes, consequently enhancing their expression [45,49,53]. The dynamic control of MMP-9-dependent H3NT proteolysis, mediated by p300/CBP-induced H3K18ac, significantly contributes to the efficient transcription of MMP-9 responsive genes in melanoma cells [49]. These findings hold substantial significance, unveiling previously undocumented functions of MMP-9 and p300/CBP, and shedding light on novel epigenetic mechanisms that propel the expression of genes involved in melanomagenesis. In a recent report, it was shown that MMP-9 is overexpressed and responsible for catalyzing H3NT proteolysis in colon cancer cells. [50]. The genome-wide transcriptome analysis showed that growth-regulatory genes are selectively targeted and activated by MMP-9-dependent H3NT proteolysis in colon cancer cells. These results unveil a previously uncharacterized function of nuclear MMP-9 and underscore the diagnostic, prognostic, and therapeutic potential of H3NT clipping to prevent the onset of colonic tumorigenesis.

## 5. Cleavage of Histones H4

The study revealed that granzyme A (GzmA) cleaves histone H4 during the apoptotic process in Raji cells (Table 1) [64]. The amount of cleaved histone H4 fragments increased in a dose-dependent manner with the caspase inhibitor in these cells, and the cleavage site was located on the histone H4 tail. Granzyme A (GzmA), an endogenous serine protease found in the cytotoxic granules of natural killer cells and cytotoxic T cells, is suggested to disrupt chromatin architecture by cleaving the H4 tail specifically in response to staurosporine treatment in Raji cells [64]. Additionally, another study demonstrated that the N-terminal tail of H4 is removed during intestinal cell differentiation by proteolytic cleavage by trypsin or chymotrypsin at residues 17-19, which reduces p-IκBα binding [65]. It has also been shown that truncation of the H4 tail affects DNA wrapping efficiency and chromatin structure, highlighting its role in regulating chromatin architecture [66]. These findings underscore the broader role of H4 tail cleavage in specific cellular differentiation processes, suggesting that the cleavage of histone tails plays a significant role in cellular function and differentiation.

## 6. Epigenetic Regulation through Histone Cleavage Mechanism

**Figure 2:**
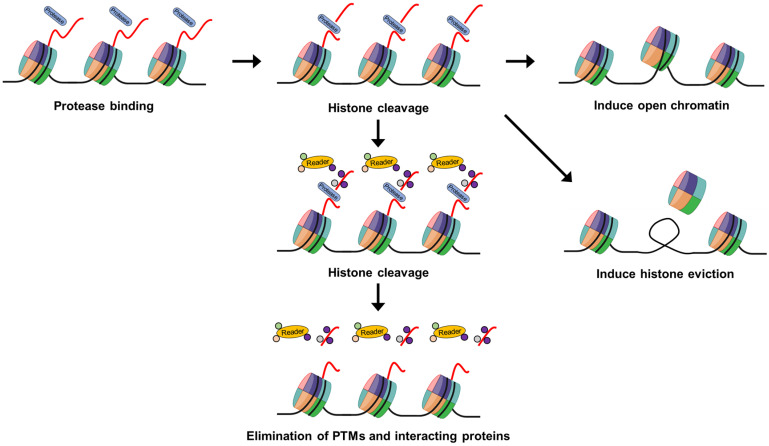
**Schematic model depicting the functional implications of histone cleavage in gene regulation.** Protease-mediated histone tail cleavages might create open chromatin structures, enhancing DNA accessibility for transcription factors and thereby promoting gene activation. Histone tail cleavage may also lead to histone eviction, providing easier access for transcription factors to DNA elements during gene activation. Additionally, histone tail cleavage might result in the extensive removal of multiple PTMs and effector ‘reader’ proteins, preventing the recruitment of associated proteins or other PTM-related processes on histone tails.

The enzymatic cleavage of histones is a pivotal process in the landscape of epigenetic modifications. As research continues to reveal an expanding repertoire of enzymes and cellular pathways involved in histone cleavage, understanding the precise mechanisms by which cleaved histones regulate gene expression remains a paramount goal in the field. Histone cleavage, characterized by the targeted proteolysis of histone proteins, represents a dynamic aspect of epigenetic regulation. This process is integral to chromatin dynamics and plays a crucial role in shaping the epigenomic landscape (Figure 2).

The discovery of novel enzymes involved in histone cleavage has provided greater understanding of this phenomenon’s intricacies. Recent studies have highlighted the roles of specific proteases, such as caspases and metalloproteases, in mediating histone cleavage events, thereby adding complexity to the regulatory network [24,40,41,42,43,47,49,50,52,55,56,63,64,67,68]. Despite advances in identifying key players in histone cleavage, the functional consequences of cleaved histones on gene expression remain a subject of intense investigation. It is increasingly evident that histone cleavage products may act as signaling entities, influencing downstream transcriptional programs [45,49,50,53]. These cleavage products, often carrying post-translational modifications, can serve as epigenetic marks that modulate the recruitment of transcriptional machinery and chromatin remodeling complexes. Furthermore, the context-dependent nature of histone cleavage adds another layer of complexity. Histone cleavage events have been observed in response to various cellular cues, including DNA damage, apoptosis, and cellular differentiation. Understanding the crosstalk between histone cleavage and these cellular processes is crucial for a comprehensive understanding of their regulatory implications.

## 7. Chromatin Dynamics and Future Directions

Eukaryotic DNA is organized into chromatin structures, requiring a compact arrangement within the nucleus. This compaction restricts the accessibility of transcription factors and RNA polymerase to gene promoters, underscoring the pivotal role of chromatin conformation in gene expression dynamics [40]. Studies employing tailless nucleosomes and nucleosomes containing truncated H3 and H4 emphasize the critical involvement of histone tails in both inter- and intra-nucleosomal interactions. Specific protease-induced truncations of histone tails enhance DNA accessibility by promoting an open nucleosome conformation, ultimately contributing to gene activation. [68,69].

Histones are evicted from gene promoters during activation, facilitating access to the transcription machinery [70,71]. In S. cerevisiae, a histone H3 endopeptidase was identified, revealing that H3 cleavage precedes histone eviction. This cleavage potentially marks nucleosomes for displacement prior to gene induction, suggesting a role for histone tail cleavage in simplifying access for the transcription machinery during gene activation [40]. Collectively, the evidence suggests a connection between histone tail cleavage and the initiation of histone eviction. Histone tails undergo PTMs at different amino acids, playing a crucial role in chromatin remodeling, DNA accessibility, and overall chromatin dynamics. Two models describe the outcomes of histone tail modifications: changes in nucleosome physical properties leading to relaxed chromatin fibers and modifications serving as recognition marks for effector protein recruitment. First, histone tail modifications can induce changes in the physical properties of the nucleosome, reducing inter- or intra-nucleosomal contacts and resulting in a more relaxed chromatin fiber [9]. Specifically, histone acetylation neutralizes the positive charge of lysine, and histone phosphorylation introduces a negative charge, both of which contribute to chromatin decondensation. Second, these modifications serve as recognition marks that facilitate the recruitment of effector proteins [9,72]. Numerous chromatin-associated factors specifically interact with modified histones through various distinct domains, including the bromodomain, chromodomain, malignant brain tumor (MBT) domain, tudor domain, plant homeodomain (PHD) finger, and PWWP domain. Various chromatin-associated factors interact specifically with modified histones through distinct domains. Although specific modifications can be removed by enzymes, histone tail cleavage emerges as an efficient method to globally erase multiple modifications, thereby impeding the recruitment of effector proteins or halting other PTM cascades on histone tails. The radical removal of various modifications through histone tail cleavage thus adds complexity to the regulatory landscape of chromatin [9,47,57,62].

Recent discoveries have revealed the extensive occurrence of histone tail cleavage across various cellular processes, identifying specific proteases responsible for these modifications. Understanding the relevance of processed histones to gene expression remains pivotal in a broader context. The findings presented in this review serve as a foundation for future investigations to delve deeper into the intricate mechanisms underpinning histone proteolysis and its profound impact on gene transcription.

Potential therapeutic implications in diseases underscore the necessity for a comprehensive exploration of the in vivo functions of histone cleavage. For instance, histone tail cleavage can significantly alter chromatin structure and gene expression, potentially enhancing the susceptibility of cancer cells to anti-cancer agents. Additionally, targeting specific proteases responsible for histone cleavage could lead to novel therapeutic strategies. These strategies might involve directly inducing histone clipping to hinder cancer cell proliferation or sensitizing cancer cells to existing treatments by modifying their epigenetic landscape [73]. Despite significant progress, there are gaps to fill; further research will likely uncover additional proteases involved in histone cleavage and reveal novel functions of clipped histones. Elucidating the detailed molecular mechanisms behind histone clipping and its direct implications in epigenetics is a crucial endeavor [74]. Recent advances have prompted intriguing questions about the complex interplay between diverse histone modifications and their role in the histone code regulating gene expression. Continued research in this area promises to enhance the understanding of epigenetic regulation and may open new avenues for medical advancements.

## Data Availability

Data contained within the article.
